# Fertility, pregnancy outcomes, and disease activity during pregnancy in patients with juvenile idiopathic arthritis: a descriptive study

**DOI:** 10.1007/s10067-025-07308-z

**Published:** 2025-01-13

**Authors:** Ali İhsan Sonkurt, Gül Doğan, Kerem Parlar, Berkay Güler, Müge Deveci, Özgür Kasapçopur, Serdal Uğurlu

**Affiliations:** 1 Cerrahpasa Medical Faculty, Istanbul University-Cerrahpasa, Istanbul, Turkey; 2 Division of Pediatric Rheumatology, Department of Pediatrics, Cerrahpasa Medical Faculty, Istanbul University-Cerrahpasa, Istanbul, Turkey; 3 Division of Rheumatology, Department of Internal Medicine, Cerrahpasa Medical Faculty, Istanbul University-Cerrahpasa, 34098 Istanbul, Turkey

**Keywords:** Biological DMARD, DMARD, Fertility, Juvenile idiopathic arthritis, Pregnancy

## Abstract

To investigate the fertility status, pregnancy outcomes, and disease activity during and after pregnancy in patients with juvenile idiopathic arthritis (JIA) currently being followed up at an adult rheumatology clinic. This study included 141 adult patients diagnosed with according to the International League of Associations for Rheumatology criteria, who are now monitored at an adult rheumatology clinic. Data on demographics, disease characteristics, medication history, fertility status, and pregnancy outcomes were collected through medical records and patient interviews. Statistical analysis was performed using Fisher’s exact test to compare categorical variables. Statistical significance was set at *p* < 0.05. Out of 141 patients (65 male, 76 female), 19 female and 6 male patients attempted to have children. Six male patients fathered ten children without assisted reproductive techniques (ART), while 16 female patients had 19 children. Only one patient couldn’t conceive. The observed pregnancy complications included miscarriage (17.4%), preeclampsia (4.3%), preterm birth (4.3%), and gestational diabetes (4.3%). Flares during or within the first month after labor in 32.2% of pregnancies. There was no statistically significant association between medication use and pregnancy complications or disease activity during pregnancy. JIA does not significantly impact fertility or increase the risk of pregnancy complications and adverse pregnancy outcomes. Disease activity during pregnancy and postpartum flares are manageable without the need for additional precautions. Patients with JIA can pursue pregnancy without significant concerns related to their condition.

**Table:** 

**Key Points** • *JIA does not significantly impact fertility in both male and female patients.* • *JIA does not increase risk of adverse pregnancy outcomes or complications.* • *JIA disease activity remains stable and manageable during pregnancy and postpartum.*

**Key Points**

• *JIA does not significantly impact fertility in both male and female patients.*

• *JIA does not increase risk of adverse pregnancy outcomes or complications.*

• *JIA disease activity remains stable and manageable during pregnancy and postpartum.*

## Introduction

Juvenile idiopathic arthritis (JIA) is defined as all forms of childhood arthritis of unknown etiology. It is the most common rheumatic disease affecting children and predominantly progresses with arthritis but can also affect extra-articular structures [1, 2]. JIA is commonly classified according to the International League of Associations for Rheumatology (ILAR) classification revised in 2001. ILAR classifies JIA into seven categories: systemic Arthritis (sJIA), oligoarthritis, polyarthritis rheumatoid factor-negative (pJIA RF-), polyarthritis rheumatoid factor-positive (pJIA RF +), psoriatic arthritis (PsA), enthesitis-related arthritis (ERA), and undifferentiated arthritis [3]. Among these subtypes, the 3 subtypes with the highest incidence and prevalence are as follows: oligoarthritis, polyarthritis, and sJIA [4]. The pharmacological treatment of JIA usually consists of DMARDs, biological agents, NSAIDs, and corticosteroids [5]. Early diagnosis and aggressive treatment are important to prevent deformities; however, the treatment used in JIA can cause long-lasting adverse effects affecting multiple systems.

There are many possible causes of impaired sexual function and reproduction in JIA, including disease-related factors and treatments [6]. Autoimmunity itself, psychological response to chronic disease, and drugs used in treatment can impair fertility ​[7]​. There are several studies regarding pregnancy outcomes and disease activity during and after pregnancy in patients diagnosed with JIA [8–16]. However, the available data regarding the fertility of JIA patients is fairly limited. Studies have reported a decreased nulliparity rate among female patients with JIA compared to references [6, 17]. Another study reported no decrease in fertility; however, there was a decrease in fecundity [18].

The maternal and fetal outcomes of patients with RA, AS, JIA, and PsA who were exposed to TNFi during pregnancy were analyzed by Genest et al. Women who stopped taking TNFi during pregnancy were at a higher risk of experiencing peripartum or postpartum flares than those who maintained treatment throughout their pregnancy [19]. Understanding the effects of different medications used in the treatment of JIA on both male and female fertility, providing guidance on discontinuing these drugs before pregnancy, and offering clear advice to patients about family planning are essential for improving routine clinical practice. In this study, we aimed to investigate the fertility status, pregnancy outcomes, and disease activity during and after pregnancy in patients with JIA currently being followed up at our adult rheumatology clinic. We also attempted to determine the effects of the drugs used in the treatment of JIA on these outcomes.

## Methods

In this study, we enrolled 141 adult patients who were previously diagnosed with JIA under the age of 16 according to the criteria of the International League of Associations for Rheumatology. These patients initially presented to our pediatric rheumatology clinic between 1990 and 2018 and are currently being followed up at our adult rheumatology clinic. Patients who presented to our adult rheumatology clinic over the age of 18 years and who exhibited disease activity were also included in the study.

The study was approved by the Istanbul University Cerrahpasa Ethical Committee (reference code: yPu62aE8), and informed consent was obtained from all participants.

Demographic characteristics, age at disease onset, disease subtype, disease severity, medication history, surgical history, prosthesis history, comorbidities, disease complications, disease duration, extraarticular features, presence of sacroillitis and the presence of autoantibodies were obtained from medical records. Data regarding the course of the disease during pregnancy and post-pregnancy were also obtained from medical records.

The patients were then thoroughly interviewed regarding their fertility status. Infertility was defined as failure to conceive after one year of regular unprotected intercourse. Marital status, duration, and number of children were recorded. Whether the patient had an infertile spouse and other possible reasons for infertility were questioned in patients with trouble conceiving. Patients with trouble conceiving were also asked whether they had used assisted reproductive techniques (ART), such as IVF. The numbers of successful and unsuccessful attempts were also noted.

Female patients who experienced pregnancy were asked additional questions regarding disease activity during and after pregnancy, and the course of pregnancy. Drugs that were used or discontinued during pregnancy were questioned. Medication doses were also recorded. First JIA flare onset after delivery and whether the patient experienced a disease flare during or the first week following birth were recorded. The duration of follow-up after birth and disease flare frequency during this period were recorded. Patient VAS scores and active joint counts before and during pregnancy, three months after birth, and at the end of the follow-up period were also recorded. Whether the patients experienced preterm birth (delivery at < 37 weeks), gestational hypertensive disorders, births with fetal anomalies, miscarriage, non-elective cesarean section, fetal growth restriction (fetal weight below the 10th percentile), gestational diabetes, and fetal asphyxia were questioned, and frequencies were noted. Male and female patients who had children and a history of using DMARDs and biological agents were questioned regarding usage duration and dose of these drugs.

Statistical analysis was performed using Fisher’s exact test to compare categorical variables and Student’s t-test to compare means between two groups. Statistical significance was set at p < 0.05.

## Results

A total of 141 patients (65 male and 76 female) were included in our study. The demographic and clinical features of the patients are shown in Tables 1 and 2, respectively. Nineteen female and six male patients attempted to have children. The demographic and clinical features of the patients who attempted to have children are shown in Table 3. All six male patients managed to have children without IVF. They had a total of ten children. One male patient had azoospermia, and his wife had a diminished ovarian reserve. They had three miscarriages before they managed to have children. All three of their pregnancies that resulted in miscarriages were achieved via IVF.

**Table 1:** Demographic features of JIA patients

*n* = 141	
Gender	
Male	n = 65 (46,1%)
Female	n = 76 (53,9%)
Age (95% confidence interval)	26.2 (25.2, 27.3)
Age at diagnosis (95% confidence interval)	9.59 (8.86, 10.34)
Education level	
Primary school	16 (11,34%)
High school	32 (22,69%)
University/College	82 (58,15%)
Unspecified	11 (7,8%)
Attempted Pregnancies	25 (17,73%)
Male Female	6 (24%) 19 (76%)
Marital status	
Married males	10 (15,38%)
Single males	55 (84,61%)
Divorced males	0 (0%)
Married females	24 (31,57%)
Single females	52 (68,42%)
Divorced females	1 (1,31%)

**Table 2:** Clinical features of JIA patients

	Female	Male	Total
Subtypes	76 *53,90%*	65 *46,09%*	141
Systemic JIA	**8** *10,52%*	**16** *24,61%*	**24** *17,02%*
Oligoarticular JIA	**21** *27,63%*	**6** *9,23%*	**27** *19,14%*
*Extended*	*5 6,57%*	*2 3,07%*	*7 4,96%*
*Persistent*	*16 21,05%*	*4 6,15%*	*20 14,18%*
Polyarticular JIA	**23** *30,26%*	**6** *9,23%*	**29** *20,56%*
*RF (* +*)*	*6 7,89%*	*0*	*6 4,25%*
*RF (-)*	*17 22,36%*	*6 9,23%*	*23 16,31%*
Psoriatic JIA	**6** *7,89%*	**4** *6,15%*	**10** *7,09%*
Enthesitis related JIA	**13** *17,1%*	**31** *47,69%*	**44** *31,2%*
Undifferentiated JIA	**5** *6,57%*	**2** *3,07%*	**7** *4,96%*
Joint Prothesis	1 *1,31%*	4 *6,15%*	5 *3,54%*
Decreased Fertility Diagnosis	2 *2,63%*	1 *1,53%*	3 *2,12%*
*PCOS*	2 *2,63%*	0	2 *1,41%*
*Azoospermia*	0	1 *1,53%*	1 *0,7%*
*Unknown Origin*	0	0	0
Infertility Diagnosis	1 *1,31%*	0	1 *0,7%*
*Prolactinoma*	1 *1,31%*	0	1 *0,7%*
*Unknown Origin*	0	0	0
Pregnancies	n = 19 *25%*	n = 6 *9,23%*	n = 25 *17,73%*
*Total Pregnancy*	25	13	38
*Live birth*	19 *76%*	10 *76,92%*	29 *76,31%*
*IVF*	0	0	0
*Natural Pregnancy*	19 *76%*	10 *76,92%*	29 *76,31%*
*Abortus*	4 *16%*	3 *23,07%*	7 *18,42%*
*IVF*	1 *4%*	3 *23,07%*	4 *10,52%*
*Natural Pregnancy*	3 *12%*	0	3 *7,89%*
*Current Pregnancy*	2 *8%*	0	2 *5,26%*

*JIA Juvenil Idiopathic Arthritis*

*RF Rheumatoid Factor*

*IVF *In Vitro* Fertilization*

*PCOS Polycystic Ovary Syndrome*

**Table 3:** Clinical and demographic features of JIA patients attempting pregnancy

	Female n = 19 *76%*	Male n = 6 *24%*	Total n = 25
Subtype			
Systemic JIA	**3 *15,78%***	**2 *33.33%***	**5 *20%***
Oligoarticular JIA	**3 *15,78%***	**0**	**3 *12%***
*Extended*	*0*	*0*	*0*
*Persistent*	*3 15,78%*	*0*	*3 12%*
Polyarticular JIA	**7 *36,84%***	**1 *16,66%***	**8 *32%***
*RF (* +*)*	*3 15,78%*	*0*	*3 12%*
*RF (-)*	*4 21,05%*	*1 16,66%*	*5 20%*
Psoriatic JIA	**1 *5,26%***	**0**	**1 *4%***
Enthesitis related JIA	**4 *21,05%***	**2 *33,33%***	**6 *24%***
Undifferentiated JIA	**1 *5,26%***	**1 *16,66%***	**2 *8%***
Age (95% confidence interval)	28.1 (26.18, 30.04)	32.66 (26.27, 39.07)	29.23 (27.19, 31.21)
Age at diagnosis (95% confidence interval)	11.53 (9.53, 13.53)	7.5 (5.43, 9.57)	10.56 (8.88, 12.24)
Patients’ education			
*Primary school*	1 *5,26%*	1 *16,66%*	2 *8%*
*High school*	7 *36,84%*	2 *33,33%*	9 *36%*
*University*	9 *47,36%*	2 *33,33%*	11 *44%*
*Education data missing*	2 *10,5%*	1 *16,66%*	3 *12%*
Marital status			
*Married*	19 *100%*	6 *100%*	25 *100%*
*Single*	0	0	0
*Divorced*	0	0	0
Decreased Fertility Diagnosis	2 *10,52%*	1 *16,66%*	3 *12%*
*PCOS*	2 *10,52%*	0	2 *8%*
*Azoospermia*	0	1 *16,66%*	1 *4%*
*Unknown Origin*	0	0	0
Infertility Diagnosis	1 *5,26%*	0	1 *4%*
*Prolactinoma*	1 *5,26%*	0	1
*Unknown Origin*	0	0	0

*Pa. No.: Patient Number*

*PCOS: Polycystic Ovary Syndrome*

*RF: Rheumatoid Factor*

*JIA: Juvenil Idiopathic Arthritis*

Out of the nineteen female patients, sixteen managed to have healthy children and they have a total of nineteen children. None of them used IVF. One of these patients had a miscarriage before having children; however, she later had two successful pregnancies. She had been diagnosed with RF + polyarticular JIA for five years. She had a previous history of MTX therapy (1 year); however, she stopped using it before starting the pregnancy attempts. She continued to use prednisolone in all three pregnancy attempts. After the first miscarriage, she started using certolizumab and switched to etanercept after the second miscarriage. She stopped using both medications while attempting to conceive. Another patient had prolactinoma; however, she managed to have a healthy child without any complications.

Of the three female patients who do not have children, two are currently pregnant. One of the pregnant patients has PCOS. Other patient, who is currently pregnant, had two prior miscarriages. The patient did not have any comorbidities known to cause reduced fertility. She had been diagnosed with ERA for seven years. She had no history of DMARD use and only used indomethacin for a total of six years but stopped six years before attempting pregnancy. The patient who does not have children or is pregnant also has PCOS. She had been diagnosed with RF-polyarticular JIA for twenty-two years. She managed to conceive once with IVF but had a miscarriage. She had a ten-year history of using methotrexate. She was treated with azathioprine (10 years), prednisolone (10 years), and adalimumab (1 year) before attempting pregnancy. Azathioprine and prednisolone were discontinued, and she continued using adalimumab during the time she tried to conceive. The patient is planning to attempt IVF to conceive again.

Eighteen female patients had a total of twenty-three pregnancies excluding the two ongoing pregnancies. Four pregnancies were complicated by miscarriage (17.4%), one with preeclampsia (4.3%), one with preterm birth (4.3%), and one with maternal diabetes (4.3%). There were ten C-Sects. (43.4%). There were no cases of fetal growth restriction, fetal anomalies, or stillbirth in our cohort. The patient with preeclampsia was diagnosed with RF- polyarticular JIA for seventeen years. She had a drug history of MTX (2 years). She was on etanercept (3 years) and prednisolone (17 years) before starting the pregnancy attempts. The patient who developed maternal diabetes had been diagnosed with undifferentiated JIA for eleven years. She had a history of etanercept (2 years) and azathioprine (2 years) use. She had been using colchicine for one year and continued to use it while trying to conceive. The patient who had a preterm delivery had been diagnosed with ERA for eleven years. The patient was also diagnosed with Behçet’s Disease. She had a history of treatment with azathioprine (6 years) and acemetacin (7 years). She stopped using etanercept after starting pregnancy attempts, which she had been using for two years. She had been on colchicine therapy for the past ten years however, she did not stop using it after starting pregnancy attempts. Pregnancy complications in relation to DMARD or biological agent exposure during conception or second and third trimesters are displayed in Table 4.

**Table 4:** Pregnancy complications in relation to DMARD or biological agent exposure during conception or second and third trimester

	Adalimumab exposure during conception or second and third trimester, *n* = 1	Hydroxychloroquine exposure during conception or second and third trimester,* n* = 2	Certolizumab exposure during conception or second and third trimester, *n* = 3	No DMARD or biological agent exposure during conception or second and third trimester, *n *= 14
Miscarriage, *n* (%)	1 (100)	0 (0)	0 (0)	3 (21.4)
Preterm birth,* n *(%)	0 (0)	0 (0)	0 (0)	1 (7.1)
Preeclampsia, *n* (%)	0 (0)	0 (0)	0 (0)	1 (7.1)
Maternal diabetes,* n* (%)	0 (0)	0 (0)	0 (0)	1 (7.1)
Non-elective c-section,* n* (%)	0 (0)	0 (0)	2 (66.7)	8 (57.1)
No complications, *n* (%)	0 (0)	2 (100)	1 (33.3)	5 (35.7)

The disease activity of JIA during pregnancy was analyzed in a total of sixteen pregnancies after excluding first-trimester miscarriages and pregnancies with insufficient data. The relationship between JIA disease activity, pregnancy, and DMARD/biological agent use is shown in Table 5. There were no statistically significant associations. Patient VAS scores and the active joint counts before and during pregnancy, three months after birth, and at the end of the follow-up period are also displayed (Table 5). Patient VAS scores were 3.6, 4.5, and 4.2, before, during, and after birth respectively. The active joint count was 3.5, 2.3, and 3.5, before, during, and after birth respectively. JIA flare during labor was observed in one pregnancy (5.3%), four (21.1%) were observed during the first week after labor, three (15.7%) were observed between the first week and first month, no flares in the first month after labor was observed in eleven (57.8%) pregnancies. The relationship between pregnancy complications, disease activity, and drug use in female patients with JIA can be seen in Table 6. There were no statistically significant associations between drug use and pregnancy complications or disease activity.

**Table 5:** Relationship between JIA disease activity, pregnancy, and DMARD/biological agent use

	DMARD or biological agent exposure during conception or second and third trimester, *n* = 5*	No DMARD or biological agent exposure during conception or second and third trimester, *n* = 11	p	Total, *n *= 16
Duration of follow-up after birth, months (95% confidence interval)	56.0 (9.0, 103.0)	38.0 (19.5, 56.5)	−	43.6 (27.1, 60.1)
JIA flare frequency per year during follow-up after birth (95% confidence interval)	1.6 (−0.3, 3.4)	2.5 (0.7, 4.3)	0.50	2.2 (1.0, 3.5)
Patient VAS score before pregnancy (95% confidence interval)	4.2 (2.0, 6.4)	3.4 (1.9, 4.8)	0.23	3.6 (2.6, 4.7)
Patient VAS score during pregnancy (95% confidence interval)	5.4 (2.1, 8.7)	4.1 (2.2, 6.0)	0.20	4.5 (3.0, 6.0)
Patient VAS score three months after birth (95% confidence interval)	4.0 (2.5, 5.5)	4.3 (2.2, 6.3)	0.43	4.2 (2.9, 5.5)
Patient VAS score at the end of follow-up (95% confidence interval)	3.6 (1.7, 5.5)	2.5 (1.0, 3.9)	0.15	2.8 (1.8, 3.9)
Active joint count before pregnancy (95% confidence interval)	4.2 (−0.8, 9.2)	3.2 (1.4, 4.9)	0.27	3.5 (1.9, 5.1)
Active joint count during pregnancy (95% confidence interval)	3.8 (−1.4, 9.0)	1.6 (0.6, 2.7)	0.07	2.3 (0.9, 3.8)
Active joint count three months after birth (95% confidence interval)	3.4 (−1.7, 8.5)	3.5 (1.1, 6.0)	0.47	3.5 (1.5, 5.5)
Active joint count at the end of follow-up (95% confidence interval)	2.8 (0.1, 5.5)	2.5 (1.3, 3.8)	0.15	2.6 (1.6, 3.6)

^*^Hydroxychloroquine in 2 pregnancies, certolizumab in 3 pregnancies

**Table 6:** Relationship between pregnancy complications, disease activity, and drug use in female patients with JIA

	DMARD or biological agent exposure during conception or second and third trimester,* n* = 5	No DMARD or biological agent exposure during conception or second and third trimester, *n* = 14	*p*-value
Preterm birth, *n *(%)	0 (0)	1 (7.1)	1.00
Preeclampsia, *n* (%)	0 (0)	1 (7.1)	1.00
Maternal diabetes, *n *(%)	0 (0)	1 (7.1)	1.00
Non-elective c-section, *n* (%)	2 (40.0)	8 (57.1)	1.00
First post-partum JIA flare onset, n (%)			
During labour	0 (0)	1 (7.1)	1.00
During first week	2 (40)	2 (14.3)	0.27
Between first week and first month	1 (20)	2 (14.3)	1.00
No flare in first month	2 (40)	9 (64.3)	0.60

## Discussion

In our study, we surveyed a group of adult patients with JIA and investigated their fertility status, pregnancy outcomes, and disease activity during and after pregnancy. Only 25 of the 141 included patients had attempted pregnancy before. This may be due to our patient's average age (26.2 ∓ 4.5) being low as we are a transition clinic. The 25 patients with prior pregnancy attempts had an average of 29.23 ∓ 4.31. The mean age at diagnosis was 10.43 ∓ 3.65. Nineteen (76%) were female and six (24%) were male. The most common subtype was polyarticular JIA (32%). The other common subtypes were enthesitis-related JIA (24%) and systemic JIA (20%). This information is important to consider when discussing the results, as the disease subtype affects the treatment modality.

JIA’s effect on fertility has been a topic of discussion. Ferreira et al. reported diminished ovarian reserve in patients with JIA [20]. However, anti-müllerian hormone (AMH) levels were not reduced, and ovarian levels were reassuring among JIA patients who used biological DMARDs in the study by Ozer et al. [21]. A study by Perez-Garcia et al. suggests that male patients diagnosed with inflammatory arthritis before or during peak reproductive years tend to have more fertility problems [22]. However, only 1.59% of patients in their cohort were diagnosed with JIA. Wallenius et al. included 75 female JIA patients and reported a decreased relative fertility rate in the JIA group compared to controls [6]. Our study is the first to report fertility rates among studies specific to JIA. In our cohort, of the 25 patients with child wish, only three don’t have children, and two of them are currently pregnant. The only patient who could not conceive had PCOS. The infertility rate in the general population is approximately 12–15% [23]. Therefore, in our experience, JIA patients did not seem to experience any fertility programs.

Research on the relationship between JIA and pregnancy outcomes and complications is more extensive than research on fertility. A 2013 study that included 78 JIA pregnancies reported increased rates of preeclampsia, preterm birth, and maternal morbidity [8]. A study conducted in 2016 reported higher rates of preterm birth, congenital malformations, and fetal growth restriction than in controls [14]. A study conducted in 2017 also reported increased rates of preeclampsia, preterm birth, and fetal growth restriction [15]. These three studies reported significant amounts of complications; however, newer studies suggest otherwise. In 2019, Drechsel et al. reported no increase in the risk of major adverse pregnancy outcomes. They reported slightly increased rates of premature birth and cesarean section compared with the German population [9]. A study conducted in 2020 only reported an increased frequency of fetal growth restriction among Asian mothers with JIA [10].

In our cohort, 18 female patients had a total of twenty-three pregnancies. Four pregnancies were complicated by miscarriage (17.4%), one with preeclampsia (4.3%), one with preterm birth (4.3%), and one with gestational diabetes (4.3%). There were no reports of stillbirth, congenital malformations, or fetal growth restriction. The Turkish population averages for miscarriage, stillbirth, preterm birth, and gestational diabetes are 13%, 1%, 12.9%, and 8.1% respectively [24–26]. The frequency of preeclampsia in the global population ranges from 2 to 10% [27]. The frequency of all complications, except miscarriage, was lower in our cohort than in the general population. The difference in miscarriage rate was also minimal (13% vs. 17.4%). This can be considered insignificant as the sample size of pregnancies in our study was low, which is a limitation of our study. These values show that JIA has no effect on pregnancy complications, and patients can conceive without any increased risk. The results of recent studies on this topic also support our results [9, 10].

Gerosa et al. included 31 women who had 49 pregnancies in their study. Disease activity significantly increased in the second trimester and nonsignificantly decreased in the third. The number of biological drugs taken and the duration of exposure to biologics during pregnancy was found to be correlated with pregnancy morbidity. Disease activity peaked three months postpartum and decreased at one year postpartum. Postpartum disease activity was found to be inversely associated with exposure to biologics post-conception [12].

In our cohort, an increase in the active joint count were not observed during pregnancy, and disease flares during or the first month following labor were observed in only 32.2% of pregnancies. None of these parameters showed a significant association with DMARD or biological agent exposure, unlike the findings of Gerosa et al. [12]. There were no statistically significant associations between patient VAS scores, active joint count, and exposure to DMARDs or biological agents. Ursin et al. also reported that nearly 80% of patients were in remission or had low disease activity during and after pregnancy, which supports our findings [13]. Therefore, pregnancy did not have a remarkable effect on disease activity and most patients did not have a disease flare in the month following birth. Additional precautions do not seem to be necessary in managing patients with JIA during or after birth.

The main limitations of our study were its retrospective nature and relatively small sample size. Its retrospective nature may have introduced recall bias while interviewing patients. We encourage future prospective studies with larger sample sizes to confirm our findings. Another limitation of our study is that we had to compare our outcomes with national data instead of a local control population, as a local control population was not available. In addition, we could not make any meaningful comparisons regarding the c-section rates as the rate of c-sections in the Turkish population is very high (60.1%), with many unnecessary procedures being performed [28].

In conclusion, our study is unique in its approach to fertility. It discusses fertility, pregnancy course, disease activity during pregnancy, pregnancy outcomes, and pregnancy complications, while other studies approach these topics more specifically. We investigated the pregnancy status of patients in our single-center transition clinic. JIA does not have a significant effect on the disease course during pregnancy, pregnancy outcomes, or complications in female patients. No condition alerted us regarding the disease activity or outcomes during pregnancy. In addition, JIA did not affect fertility in either males or females. Therefore, patients with JIA can try to conceive without any worries, and additional precautions do not seem to be necessary during follow-up.

## Data Availability

All data relevant to the study are included in the article.

## Declarations

The study complies with the Declaration of Helsinki. The research protocol has been approved by the locally appointed ethics committee.
